# Intramyocardial Injection of Hypoxia-Conditioned Extracellular Vesicles Modulates Response to Oxidative Stress in the Chronically Ischemic Myocardium

**DOI:** 10.3390/bioengineering11020125

**Published:** 2024-01-28

**Authors:** Dwight D. Harris, Sharif A. Sabe, Mark Broadwin, Cynthia Xu, Christopher Stone, Meghamsh Kanuparthy, Akshay Malhotra, M. Ruhul Abid, Frank W. Sellke

**Affiliations:** Division of Cardiothoracic Surgery, Department of Surgery, Cardiovascular Research Center, Rhode Island Hospital, Alpert Medical School of Brown University, Providence, RI 02903, USA; ddharris@bidmc.harvard.edu (D.D.H.); ssabe@bidmc.harvard.edu (S.A.S.); mbroadwin@lifespan.org (M.B.); cxu2@lifespan.org (C.X.); christopher_stone@brown.edu (C.S.); meghamsh_kanuparthy@brown.edu (M.K.); akshay_malhotra@brown.edu (A.M.); ruhul_abid@brown.edu (M.R.A.)

**Keywords:** extracellular vesicles, hypoxia-conditioned extracellular vesicles, swine, chronic myocardial ischemia, oxidative stress

## Abstract

Introduction: Patients with advanced coronary artery disease (CAD) who are not eligible for stenting or surgical bypass procedures have limited treatment options. Extracellular vesicles (EVs) have emerged as a potential therapeutic target for the treatment of advanced CAD. These EVs can be conditioned to modify their contents. In our previous research, we demonstrated increased perfusion, decreased inflammation, and reduced apoptosis with intramyocardial injection of hypoxia-conditioned EVs (HEVs). The goal of this study is to further understand the function of HEVs by examining their impact on oxidative stress using our clinically relevant and extensively validated swine model of chronic myocardial ischemia. Methods: Fourteen Yorkshire swine underwent a left thoracotomy for the placement of an ameroid constrictor on the left circumflex coronary artery to model chronic myocardial ischemia. After two weeks of recovery, the swine underwent a redo thoracotomy with injection of either HEVs (n = 7) or a saline control (CON, n = 7) into the ischemic myocardium. Five weeks after injection, the swine were subjected to terminal harvest. Protein expression was measured using immunoblotting. OxyBlot analysis and 3-nitrotyrosine staining were used to quantify total oxidative stress. Results: There was a significant increase in myocardial expression of the antioxidants SOD 2, GPX-1, HSF-1, UCP-2, catalase, and HO-1 (all *p* ≤ 0.05) in the HEV group when compared to control animals. The HEVs also exhibited a significant increase in pro-oxidant NADPH oxidase (NOX) 1, NOX 3, p47phox, and p67phox (all *p* ≤ 0.05). However, no change was observed in the expression of NFkB, KEAP 1, and PRDX1 (all *p* > 0.05) between the HEV and CON groups. There were no significant differences in total oxidative stress as determined by OxyBlot and 3-nitrotyrosine staining (*p* = 0.64, *p* = 0.32) between the groups. Conclusions: Administration of HEVs in ischemic myocardium induces a significant increase in pro- and antioxidant proteins without a net change in total oxidative stress. These findings suggest that HEV-induced changes in redox signaling pathways may play a role in increased perfusion, decreased inflammation, and reduced apoptosis in ischemic myocardium. Further studies are required to determine if HEVs alter the net oxidative stress in ischemic myocardium at an earlier time point of HEV administration.

## 1. Introduction

As the leading cause of morbidity and mortality worldwide, ischemic heart disease remains a pervasive global health challenge [1,2]. Despite advances in medical science, the therapeutic options available for the treatment of heart disease are still limited, underscoring the urgent need for the development of novel and effective treatments [3,4]. Current interventions such as medical management, stenting, and bypass surgery are aimed at the management of symptoms and the prevention of further damage, rather than the restoration normal myocardial function [5]. As the prevalence of cardiovascular disease continues to grow with an aging population, the pursuit of novel therapeutics is essential to augment the options available for patients with end-stage disease, for whom merely temporizing measures are not sufficient [6,7].

Extracellular vesicles (EVs) have emerged as a promising therapeutic modality for the treatment of heart disease in this setting [8,9,10]. In the course of normal physiology, these nanosized vesicles are released by various cell types into the extracellular environment, playing a crucial role in cell-to-cell communication [11,12,13]. Differentiated according to size into exosomes, microvesicles, and apoptotic bodies, EVs carry a cargo of proteins, lipids, and nucleic acids that reflects the physiological state of their parent cells [13]. They participate in diverse biological processes, including immune modulation, angiogenesis, and tissue repair [13,14,15]. EVs have demonstrated potential in a multitude of disease processes, including limb ischemia, trauma, and ischemic cardiovascular disease [9,16,17,18,19]. In normal biological process, it appears that EVs can promote both beneficial as well as destructive cellular processes [20,21]. The ex vivo engineering and injection of EVs appears to have overall beneficial effects [22,23]. Rodent models of cardiac disease have yielded modulation of cardiac inflammation and augmented cardiac function with EV treatment [10,24]. Our group has identified several promising changes using EVs derived from human bone-marrow-derived stem cells (HBMSCs) in our validated swine model of chronic myocardial ischemia with and without concomitant metabolic syndrome [25,26]. Using this model, we have demonstrated increased angiogenesis and cardiac function, decreased inflammation, and modulation of angiogenic signaling [25,26].

EVs are conventionally developed in conditions featuring atmospheric oxygen levels and serum deprivation [27]. Nevertheless, it is possible to enhance the characteristics and therapeutic capabilities of EVs through preconditioning [27,28,29,30]. Particularly noteworthy in this regard is the application of hypoxia conditioning, whereby EVs are adapted to perform effectively in oxygen-deprived settings. This is of particular interest in cardiovascular disease, as EVs can be modified to function optimally in the ischemic myocardium found in advanced coronary artery disease (CAD). Previous research has demonstrated that hypoxia-conditioned extracellular vesicles (HEVs) exhibit a capacity to reduce both overall apoptosis and infarct size in a rodent model of reperfusion injury [29]. Our laboratory has developed and extensively studied a line of HEVs, with which we have previously demonstrated that non-starved HEVs express the largest number of sub-proteins when compared with normoxic non-starved EVs, normoxic serum-starved EVs, and serum-starved HEVs [27]. We have previously published proteomic, immunoblotting, and electron-microscopy-generated morphologic data that demonstrated many changes, including in metabolic pathways, redox proteins, and in angiogenesis [27]. Given both our prior success with standard, normoxic EVs and the potential benefits of HEVs, our lab set out to test HEVs with a validated large animal (porcine) model of chronic myocardial ischemia [25,26]. With this model, we have shown reductions in apoptosis and myocardial inflammation as well as improved myocardial perfusion using HEVs [31,32,33]. The goal of this study is to extend these insights through an improved understanding the of the molecular underpinnings of the findings previously derived from our model, with a particular emphasis on oxidative stress pathways.

## 2. Methods

### 2.1. Large Animal Swine Model

**Figure 1:**
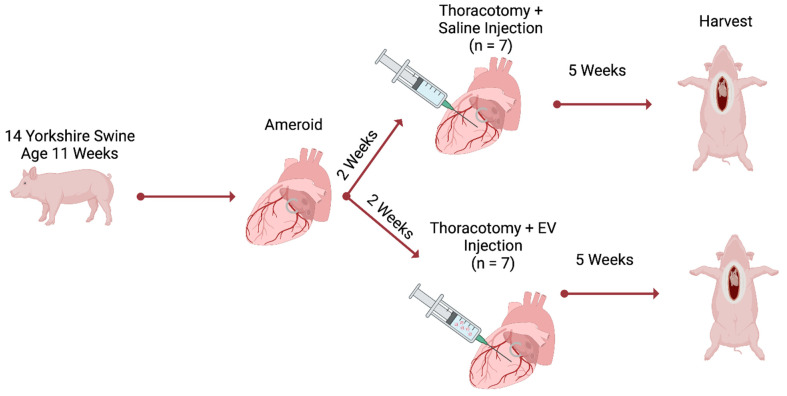
Methods. Chronic myocardial ischemia was modeled by placement of an ameroid constrictor on the left circumflex coronary artery. The ameroid was allowed to close for two weeks; after which, swine underwent a redo left thoracotomy for intramyocardial injection of either hypoxia-conditioned extracellular vesicles (EV, n = 7) or normal saline (n = 7). Five weeks after injection, swine underwent terminal harvest.

This study is a follow-up analysis of our previously published cohort of fourteen Yorkshire swine (Cummings School of Veterinary Medicine of Tufts University Farm, Grafton, MA, USA) [32]. Chronic myocardial ischemia was modeled by placement of an ameroid constrictor (Research Instruments SW, Lebanon, OR, USA) around the left circumflex coronary artery (LCx). The ameroid was allowed to close for two weeks; after which, swine underwent redo left thoracotomy with intramyocardial injection of either HEVs (n = 7, male = 3, female = 4) or normal saline (CON, n = 7, male = 3, female = 4) [32]. Swine then underwent a final terminal harvest five weeks after injection (Figure 1).

### 2.2. Humane Animal Care

All animals received humane care as previously reported and in compliance with the *Guide for the Care and Use of Laboratory Animals* [32]. All experiments were approved by the Institutional Animal Care and Use Committee of Rhode Island Hospital (approval number: 1791190-26).

### 2.3. Ameroid Placement

Swine received cephalexin (30 mg/kg) and aspirin (10 mg/kg) orally 1-day preoperatively and 5-days postoperatively. Anesthesia was induced with intramuscularly injected xylazine (2.2 mg/kg) and telazol (4.4 mg/kg). The swine were intubated, and anesthesia was maintained on inhaled isoflurane. The swine were positioned in the right lateral decubitus position with a slight tilt toward the surgeon and prepared with betadine for antisepsis [32]. A left thoracotomy was then performed in the second intercostal space [32]. The LCx was exposed using a combination of sharp and blunt dissection [32]. The dissection was carried back to the branch of the LCx from the left main coronary artery shortly after its emanation from the aortic root. The LCx was encircled with a vessel loop and temporarily occluded for a duration of two minutes; simultaneously, a 5 mL solution of gold microspheres (BioPal, Worcester, MA, USA) was injected into the right atrium to map the area at risk for ischemia secondary to ameroid-induced occlusion. Following this two-minute interval, the ameroid constrictor was positioned at the LCx base as close to the left main coronary artery as feasible, as this ensures uniform infarcts across all experimental subjects. Finally, the pericardium was irrigated with saline and closed with absorbable sutures and the chest was closed in layers using absorbable suture [32]. Postoperative pain was controlled with a fentanyl patch (4 μg/kg) placed just before surgery and buprenorphine (0.03 mg/kg) intramuscular injection before closure.

### 2.4. Extracellular Vesicle Culture

HBMSCs (Lonza, Cambridge, MA, USA) were cultivated in accordance with manufacturer’s recommendations in growth medium (MSCGM Bulletkit PT-3001; Lonza, Cambridge, MA, USA) as previously described [27,31]. The cells were cultured to 80% confluence and then passaged to passage seven. At this stage, the culture medium was exchanged with fresh MSCGM media and hypoxia was induced in the cells by incubating for 24 h at 37 °C in a humidified hypoxia chamber with an atmosphere comprised of 5% carbon dioxide and 95% nitrogen [27,31]. After 24 h, the media were collected and centrifuged at 2000× *g* twice. This was performed to remove cells and debris [27]. The extracted media were centrifuged at 100,000× *g* for 70 min to isolate the HEVs [27]. The product was then washed twice with phosphate-buffered saline and underwent an additional 70 min centrifuge cycle at 100,000× *g* [27]. The resultant HEVs were suspended in phosphate-buffered saline with 1% dimethyl sulfoxide [27]. The HEVs are stored at −80 Celsius [27]. The Pierce™ BCA Protein Assay Kit (Kit 23225; Thermo Fisher Scientific, Waltham, MA, USA) was used for protein quantification to ensure a standardized dosage for injection [27].

### 2.5. Extracellular Vesicle Proteomics

The HEV samples were prepared as described above and proteomics was conducted in triplicate (n = 3) [27]. The samples were prepared and then run on LC-MS/MS as previously described [27]. Peptide spectrum matching was searched against the UniProt protein database using the Sequest algorithm within Proteome Discoverer v 2.3 software (Thermo Fisher Scientific, Waltham, MA, USA) as previously described [27]. Relative label-free quantitative analysis was then performed using the Minora algorithm as previously described [27]. Pathway analysis was performed using ShinyGO 0.76 (South Dakota State University, Brookings, SD, USA).

### 2.6. Extracellular Vesicle Injection

HEVs (50 μg) were thawed and suspended in 2 mL of 0.9% sterile saline on the day of injection [32]. Anesthesia and preoperative care were the same as described above in Section 2.3. The swine were again placed in the modified right lateral decubitus position and prepared with betadine-based antiseptic solution [32]. A redo left thoracotomy was performed one intercostal space below the prior incision to optimize ease of surgical entry. Pulmonary adhesions were taken down with a combination of sharp and blunt dissection. The pericardium was opened. The HEVs were injected into the myocardium in ten locations adjacent to the LCx [31]. The pericardium was irrigated with saline and closed with absorbable sutures and the chest was closed in layers using absorbable [31]. Post-operative management was the same as described above in the Section 2.3.

### 2.7. Harvest and Perfusion

Anesthesia and preoperative care were the same as described above in the ameriod section. The swine were positioned supine on the operating room table and prepped with betadine, whereupon the chest was opened through a median sternotomy for maximal exposure. The pericardium was opened and the heart was dissected free from adhesions as needed. Care was taken to expose the left atrium, right atrium, and apex to facilitate subsequent steps involving placement of transduction equipment in these locations. A right groin cutdown was concurrently performed as previously described to access the right femoral artery. The artery was canulated with a 7 French vascular sheath using the Seldinger technique. The vascular sheath was connected to a withdrawal pump (Harvard Apparatus, Holliston, MA, USA). Myocardial perfusion was measured by injection of labeled microspheres (BioPAL, Worcester, MA, USA) at rest and during rapid pacing at 150 bpm to stimulate myocardial stress, while simultaneously withdrawing 10 mL of blood from the femoral artery; myocardial perfusion was also measured at rest and during rapid pacing of the heart [31,32]. A pressure volume catheter (Transonic, Ithica, NY, USA) was placed in the left ventricle using the Seldinger technique. After collection of physiologic measurements, the animals were euthanized and the heart was removed and sectioned into 16 segments based on the proximity of the tissue to the left anterior descending and LCx artery. Segments were individually stored and snap frozen in liquid nitrogen. Given the distribution of myocardial supply by the LCx coronary artery, the free wall sections were usually the most ischemic, but this was confirmed by measuring of gold microsphere concentrations to ensure that the testing was performed on the most appropriate myocardial regions.

### 2.8. Lysate Production

Lysates used in both immunoblotting and Oxybloting were produced from the ischemic myocardial tissue of seven control animals and seven experimental animals. The tissue was lysed using RIPA Lysis and Extraction Buffer (Boston Bioproducts Milford, MA, USA), the Halt Protease Inhibitor Cocktail (Thermo Fisher Scientific, Waltham, MA, USA), and an ultrasonic homogenizer [32]. The Pierce™ BCA Protein Assay Kit (Thermo Fisher Scientific, Waltham, MA, USA) was used to measure the protein concentrations of all lysates in order to ensure homogenous loading [32].

### 2.9. Immunoblotting

A total of 40 μg of protein lysate was run on a 4% to 12% Bis-Tris gel (Thermo Fisher Scientific, Waltham, MA, USA) at 200 volts for 40 min and then transferred onto a nitrocellulose membrane (Bio-Rad, Hercules, CA, USA) in a room set to 4 °C at 40 volts for 14 h [32]. The membrane was then blocked in a 5% non-fat dry milk solution in tris-buffered saline (TBST, Boston BioProducts, Milford, MA, USA) for 1 h [32]. The membranes were mixed with primary antibody dilutions in 3% bovine serum albumin in TBST and set to incubate in the 4 °C room for 24 h. The membranes were then removed from the cold room and incubated in horseradish-peroxidase-linked secondary antibody dilutions in 3% bovine serum albumin in TBST for 1 h at room temperature [32]. The membranes were incubated with the Enhanced Chemiluminescence Western Blotting Substrate (Thermo Fisher Scientific, Waltham, MA, USA) developing agent and imaged on a ChemiDoc Imaging System (Bio-Rad, Hercules, CA, USA). Finally, Restore PLUS Western Blot Stripping Buffer (Thermo Fisher Scientific, Waltham, MA, USA) was used to strip membranes for repeat probing if needed for the investigation of additional targets [32]. Immunoblot band intensity was measured using Image J software version 1.54 (National Institutes of Health, Bethesda, Maryland, USA).

### 2.10. Antibodies

Primary antibodies were obtained from Cell Signaling Technology, Abcam, Protein Technology, and Novus Biologicals and incubated for 24 h in the dilutions listed in Table 1. Horseradish-peroxidase-linked secondary antibodies to mouse, rabbit, and goat were obtained from Cell Signaling Technology and Abcam and incubated for 1 h at room temperature using the dilutions listed in Table 1.

### 2.11. Oxyblot

The MilliporeSigma™ OxyBlot™ Protein Oxidation Detection Kit (Burlington, MA, USA) was used for Oxyblot analysis. A total of 20 μg of protein lysate was run on a 4% to 12% Bis-Tris gel (Thermo Fisher Scientific, Waltham, MA, USA) after mixing the lysate with the kit components in concordance with manufacturer recommendations. The gel was transferred onto a nitrocellulose membrane (Bio-Rad) in the 4 °C room at 40 volts for 14 h. The membrane was incubated with the primary and secondary antibodies provided in the Oxyblot kit at room temperature per the manufacturer’s recommendations. The membrane was incubated with the Enhanced Chemiluminescence Western Blotting Substrate (Thermo Fisher Scientific, Waltham, MA, USA) developing agent and imaged on a ChemiDoc Imaging System (Bio-Rad, Hercules, CA, USA) as above. Band intensity was measured using Image J software version 1.54 (National Institutes of Health, Bethesda, Maryland, USA).

### 2.12. Nitrotyrosine Staining

Nitrotyrosine (3NT, Table 1) staining was performed on frozen slides made from ischemic myocardial tissue from six control animals and seven experimental animals. The samples were derived from an area similar to that which was used for lysate production. The samples were stained and imaged by iHisto (Salem, MA, USA). 3NT staining was analyzed using QuPath software. Three 10 mm^2^ sections were selected per slide and analyzed using the QuPath automated detection algorithm as previously described. The average amount of 3NT positivity was calculated per section and averaged across sections.

### 2.13. Statistics

Data analysis was conducted with Prism 9 (GraphPad Software, San Diego, CA, USA). Data were analyzed for normality using the Shapiro–Wilk test. The Wilcoxon rank-sum was used to analyze nonparametric data and Student’s *t*-test was used to analyze data that followed a normal distribution. All data are represented as mean plus or minus standard deviation. Immunoblot data are represented as mean fold change normalized to the average control as previously described. The Spearman rank correlation coefficient was used to correlate protein expression and our previously reported myocardial perfusion data. Outlier analysis was conducted and data points greater than 2 standard deviations from the mean were excluded from analysis. *p*-values less than or equal to 0.05 were set as significant prior to analysis.

## 3. Results

### 3.1. Extracellular Vesicle Proteomics

The HEVs produced had a mean particle size of 179.2 ± 7.3 nm, as previously reported [27]. We have previously demonstrated the presence of the transmembrane proteins CD81 and CD91 and absence of albumin in the HEVs using immunoblotting [27]. Proteomic analysis of the HEVs identified 395 unique proteins as previously reported by our group [27]. This included 16 proteins with known antioxidant function, including annexin a1, catalase, ferritin heavy chain 1, glutathione s-transferase pi 1, heat shock protein b1, hemoglobin subunit alpha 2, hemoglobin subunit epsilon 1, peroxiredoxin-1, peroxiredoxin-2, peroxiredoxin-5, peroxiredoxin-6, phosphoglycerate Kinase 1, protein disulfide-isomerase, ras-related protein rap-1b, superoxide dismutase 1, and thioredoxin (Table 2).

### 3.2. Functional Data

**Figure 2:**
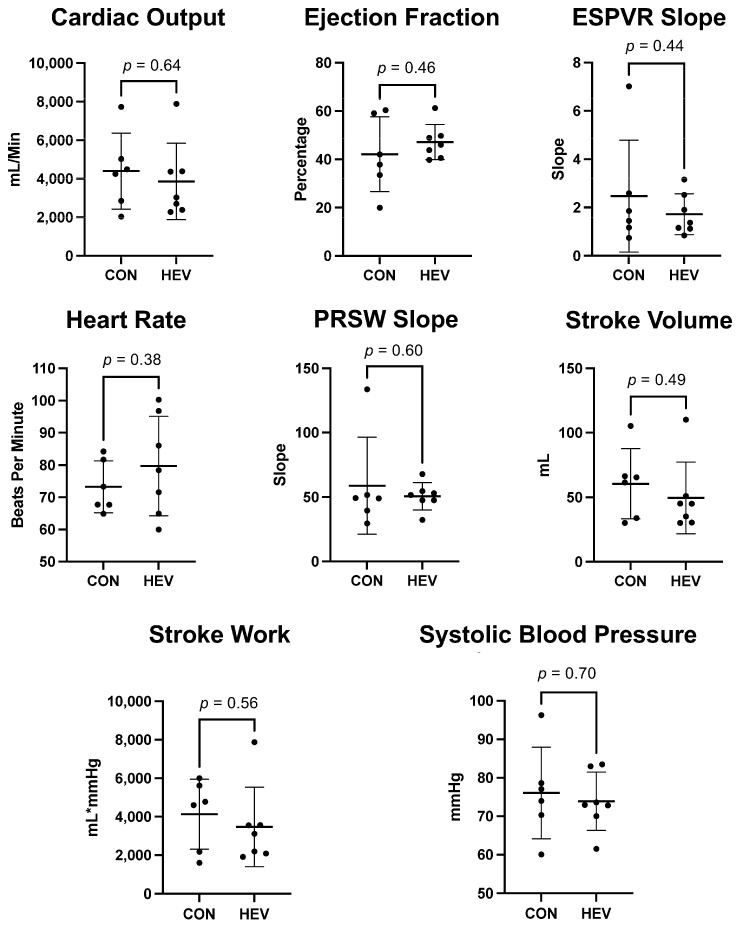
Myocardial functional data. There was no significant difference in heart rate or blood presure between the hypoxia-conditioned extracellular vesicle (HEV) group and the control group (CON). There was no significant difference in markers for systolic and diastolic function including cardiac output, stroke volume, stroke work, ejection fraction, preload recruitable stroke work (PRSW) slope, and end-systolic pressure–volume relation (ESPVR) slope between the HEV group and CON.

There was no significant difference in heart rate or blood pressure between the HEV group and CON. There was no significant difference in markers for systolic function including cardiac output, stroke volume, stroke work, and ejection fraction between the HEV group and CON (all *p* > 0.05). There was no significant change in key markers for ventricular relaxation including preload recruitable stroke work (PRSW) slope and end-systolic pressure–volume relation (ESPVR) slope between the HEV group and CON (Figure 2).

### 3.3. Myocardial Protein Expression

**Figure 3:**
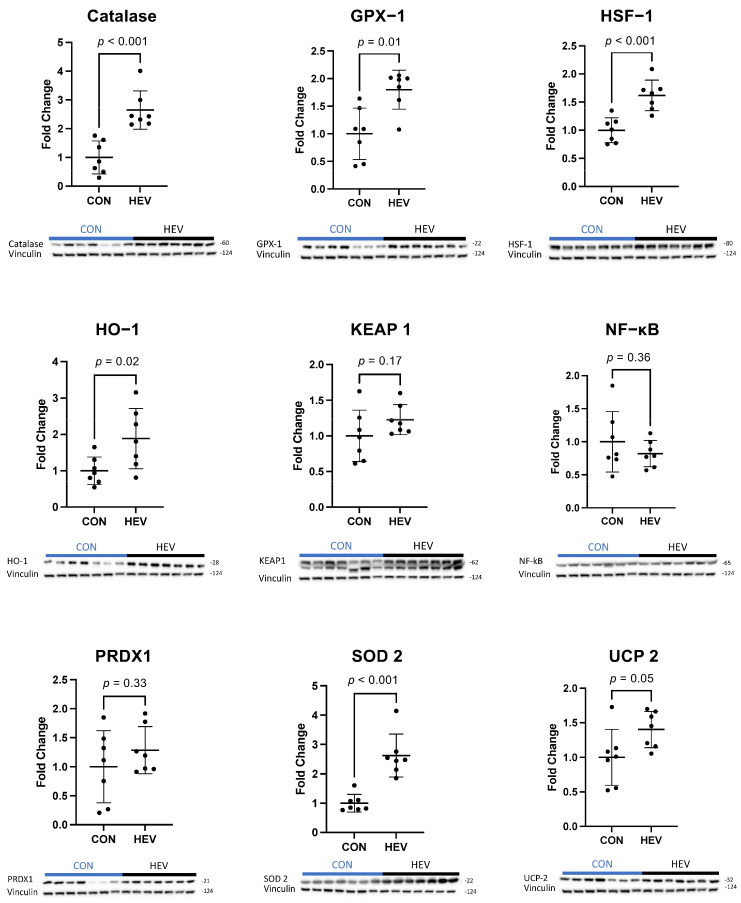
Ischemic myocardial antioxidant protein expression. There was a significant increase in antioxidant expression in the hypoxia-conditioned extracellular vesicle (HEV) group when compared to the control group (CON); increased antioxidant targets included catalase, glutathione peroxidase-1 (GPX-1), heat shock factor 1 (HSF-1), heme oxygenase-1 (HO-1), superoxide dismutase 2 (SOD 2), and mitochondrial uncoupling protein 2 (UCP-2) (all *p* ≤ 0.05). There was no change in kelch-like ECH associated protein 1 (KEAP 1), NF-kB, or peroxiredoxin-1 (PRDX1).

**Figure 4:**
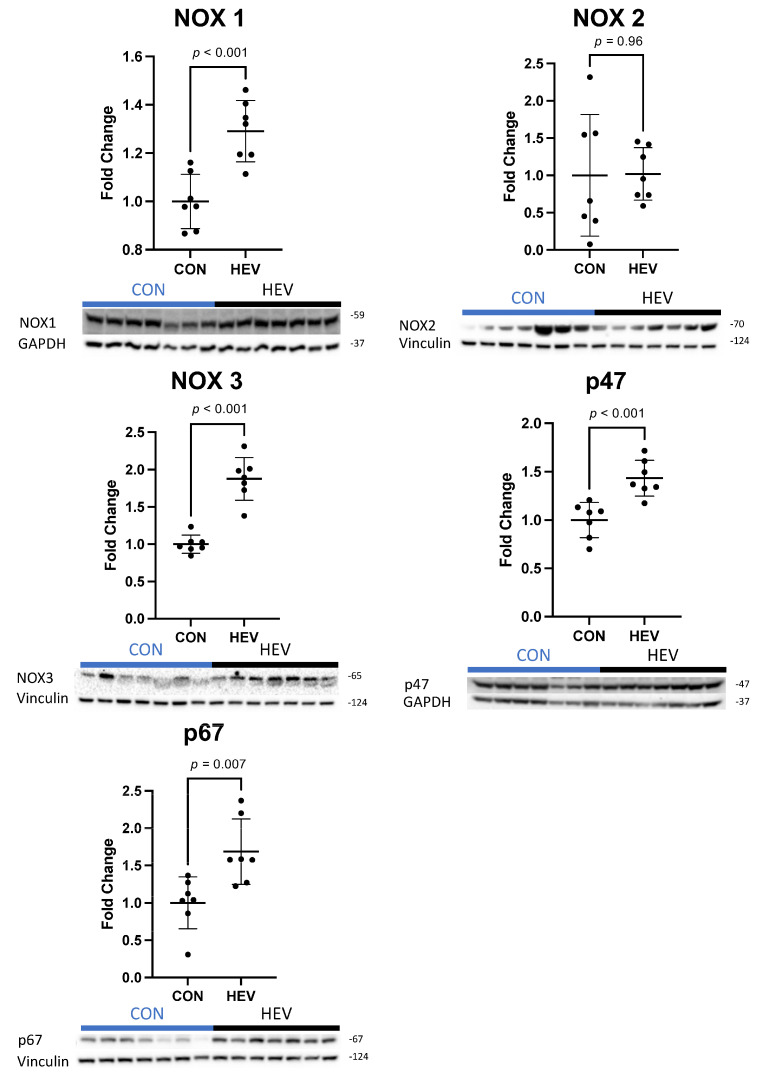
Ischemic myocardial pro-oxidant protein expression. There was a significant increase in pro-oxidant protein expression in the hypoxia-conditioned extracellular vesicle (HEV) group compared to the control group (CON), including NADPH oxidase 1 (NOX 1), NADPH oxidase 3 (NOX 3), p47phox, and p67phox. There was no expressional change in the pro-oxidant NADPH oxidase 2 (NOX 2).

There was a significant increase in the expression of several antioxidant proteins in the ischemic myocardium of the HEV group when compared to control animals, including catalase, glutathione peroxidase-1 (GPX-1), heat shock factor 1 (HSF-1), heme oxygenase-1 (HO-1), superoxide dismutase 2 (SOD 2), and mitochondrial uncoupling protein 2 (UCP-2, all *p* ≤ 0.05) (Figure 3). There was also a significant increase in the expression of the pro-oxidant proteins NADPH oxidase 1 (NOX 1), NADPH oxidase 3 (NOX 3), p47phox, and p67phox in the ischemic myocardium of the HEV group when compared to control animals (all *p* ≤ 0.05, Figure 4). There was no change in the expression of the pro-oxidant NADPH Oxidase 2 (NOX 2); nor was there any change in expression of the antioxidants NF-kB, kelch-like ECH associated protein 1 (KEAP 1), and peroxiredoxin-1 (PRDX1) between the two groups (all *p* > 0.5).

### 3.4. Myocardial Oxyblot and Nitrotyrosine Staining

**Figure 5:**
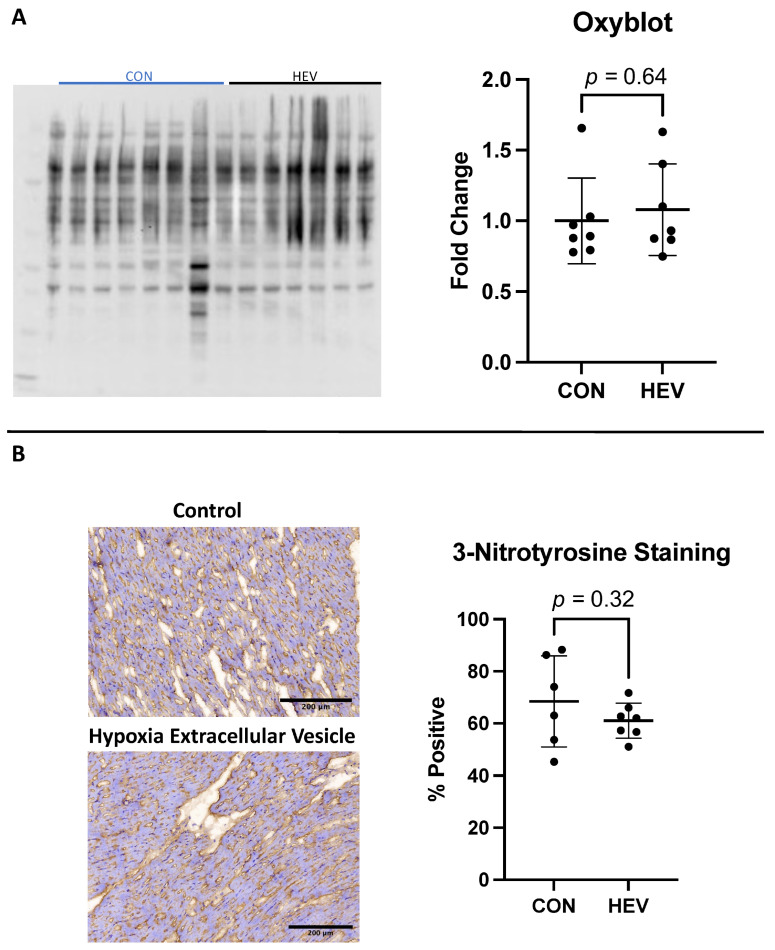
Oxyblot and 3-nitrotyrosine staining. (**A**) There was no significant difference in total oxidative stress as measured with using the Oxyblot technique between the hypoxia-conditioned extracellular vesicle (HEV) group and the control group (CON). (**B**) There was also no significant difference in total oxidative stress as measured histologically with 3-nitrotyrosine staining.

There was no significant difference between the HEV and CON groups in total oxidative stress as measured using the Oxyblot assay (*p* = 0.64, Figure 5A). There was also no significant difference between the groups in total oxidative stress when measured histologically using 3NT staining (*p* = 0.34, Figure 5B).

### 3.5. Fibrosis

**Figure 6:**
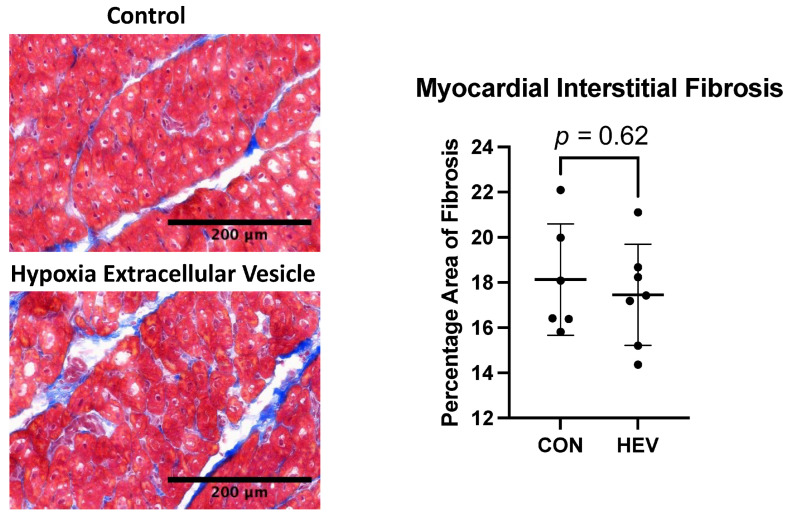
Masson’s trichrome staining. There was no significant difference in interstitial fibrosis as measured using Masson’s trichrome stain between the hypoxia-conditioned extracellular vesicle (HEV) group and the control group (CON).

There was no significant difference in interstitial fibrosis between the HEV and CON groups as measured by Masson’s trichrome stain (*p* = 0.62, Figure 6).

### 3.6. Myocardial Perfusion Correlation

Myocardial protein expression from proteins with significant changes were plotted against our previously published perfusion data for the treated myocardium [31]. There was no correlation between myocardial perfusion at rest or with passing at 150 beats per minute and ischemic myocardial protein expression (Table 3).

## 4. Discussion

EVs have emerged as a promising therapeutic strategy for the treatment of heart disease, and EV conditioning has the potential to enhance their therapeutic efficacy [9,10,30]. Using proteomic analysis, we have previously demonstrated that hypoxia-non-starved EVs, such as those used in this study, exhibit the greatest diversity in protein contents; changes noted included proteins involved in cellular metabolism, calcium handling, and the oxidative stress response [27]. In this study, we identified 16 different proteins in our hypoxia EVs (HEVs) that have been shown to modulate the response to oxidative stress. This information prompted us to hypothesize that our HEVs would induce modulation of oxidative stress in the myocardial tissue of our clinically relevant swine model.

Oxidative stress plays a crucial role in cardiovascular disease, contributing to the exacerbation of vascular injury and the progression to heart failure in patients with myocardial ischemia [34,35,36,37]. We have previously demonstrated increased myocardial perfusion, reduced apoptosis, and decreased inflammation with HEVs in our swine model of chronic myocardial ischemia [31,32,33]. This study was designed to provide a more elaborate account of these encouraging findings and represents our first attempt to understand how the intramyocardial injection of HEVs modulates the response to oxidative stress.

There was no significant change in key cardiac functional parameters including heart rate, blood pressure, cardiac output, stroke volume, stroke work, ejection fraction, PRSW slope, and ESPVR slope between the HEV group and CON. There was no change in myocardial fibrosis between the HEV group and CON. There were several key changes in myocardial response to oxidative stress, and this is likely related to our prior findings of increased perfusion, reduced apoptosis, and decreased inflammation with HEVs.

The HEV group exhibited upregulation of several key antioxidants, including catalase, GPX-1, HO-1, HSF-1, SOD 2, and UCP-2. Catalase is of particular interest, as it was also present in the proteomic analysis of EVs previously conducted by our group and thus repents a direct relationship between the EV contents and cellular changes. Other proteins that exhibited expressional changes with corollaries in our prior proteomic analysis of injected EVs included HSF-1 and HO-1. SOD 2 and the NOX complexes, by contrast, were not found in our EV proteomic analysis. Additionally, we observed proteins that are expressed in the HEVs but that are not seen in the myocardium, such as PRDX1. This suggests that, while some of the changes from our EVs are likely related to the injection of proteins, others are likely attributable to more complex cellular changes in the microenvironmental milieu, potentially involving RNA epigenetic mechanisms such as DNA methylation.

In concordance with our finding that the total oxidative damage did not differ between groups, catalase has been shown to play an important role in the modulation of reactive oxygen species and improved myocardial remodeling independent of oxidative stress [38]. GPX-1 and HO-1 are both essential antioxidants in the myocardial response to ischemia and function mechanistically as part of the Nrf2 pathway [39]. GPX-1 deficiency has been linked to cardiac hypertrophy and dysfunction, while increased expression of HO-1 has been shown to decrease ischemic myocardial injury [40,41]. HSF-1 over-expression has been linked to decreased cardiac fibrosis and cardiac dysfunction in pressure-overload-induced injury [42]. Finally, SOD 2 and UCP-2 are both antioxidants that have been shown to attenuate myocardial ischemic injury but are not clear components of the Nrf2 pathway [43].

In addition to its augmented antioxidant expression, the HEV group also exhibited significant increases in several pro-oxidant enzymes, including NOX 1, NOX 3, p47phox, and p67phox. P47phox (also known as neutrophil cytosolic factor 1) and p67phox are cytosolic subunits of the NOX 2 complex that respond to cellular activation signals, such as those provided by inflammation, by translocating to the cell membrane along with additional components for the effectuation of enzymatic functions [44]. This suggests that, although we did not observe a significant increase in NOX 2 expression, enzymatic function, and thus production of ROS in cells expressing NOX 2, may have nevertheless been increased in the group. The upregulation of NOX 1, NOX 2, and NOX 3 is generally believed to promote myocardial injury; increased NOX isoform expression has also been linked to more severe ischemic injury and maladaptive myocardial remodeling [45,46].

Notably, these adverse outcomes are directly at odds with the increase in myocardial perfusion and decrease in apoptosis in response to HEVs found in our previous study [31,32]. This discrepancy requires reconciliation, which was provided by our assays for total oxidative stress. We observed no changes in total oxidative damage using OxyBlot analysis or by 3-nitrotyrosine stanning. At the molecular level, these findings suggest that the oxidative stress generated by NOX 1, NOX 2, and NOX 3 was plausibly neutralized by the observed upregulation of the antioxidants catalase, GPX-1, HO-1, and SOD 2. This balancing of the net oxidative stress by HEV treatment may reflect an initial elevated level of oxidants in the ischemic tissue that is counteracted by HEVs that induce antioxidant signaling molecules such as catalase, GPX, and HSF-1 and results in myocardial recovery. In other words, these findings appear to suggest that modulation of oxidative signaling pathways, both pro- and antioxidant, by HEVs produce favorable myocardial functional effects independently of any net effects on tissue oxidative stress.

In conclusion, the intramyocardial injection of HEVs results in a complex modulation of redox signaling pathways within the ischemic myocardium. HEV injection was linked to an increase in several key antioxidants, such as catalase, GPX-1, HO-1, HSF-1, SOD 2, and UCP-2, as well as to upregulation of several markers of pro-oxidant signaling, such as NOX 1, NOX 2 subunit, and NOX 3. This complex interplay resulted in no overall change in tissue-level oxidative stress, but the upregulation of antioxidants appeared to have potentially played a critical role in adaptive remodeling and in the improvements in cardiac function by HEVs.

This study furthers our understanding of the use of conditioned EVs in the treatment of ischemic heart disease. It does, however, have several important limitations. First, given the constraints imposed by its design incorporating a large animal model, the sample size of the study is relatively small: this may render it underpowered to detect subtle changes. Additionally, heart disease is known to be a sex-specific illness and, although our design incorporated a mix of male and female animals, the small sample size precludes the performance of meaningful sex-specific subgroup analyses. Beyond considerations related to sample size, the design of our study is limited by the use of a single time point (at five weeks after HEV administration) for data collection following administration of EV therapy, as it is plausible that HEVs exert their effects in a manner that builds or wanes as further time passes from their administration. This study was not designed to look at fresh tissue and as such we did not have ability to conduct enzyme function assays. Finally, EV therapy is limited in general by the variability in EV production and contents across different labs [47]. This is an important concern among research teams that study EVs and will require rectification to optimize the ease of large-scale EV production and distribution [47].

## 5. Conclusions

Intramyocardial injection of HEVs has been linked to decreased myocardial injury and improved myocardial function. This study demonstrates that injection of HEVs results in a complex modulation of redox signaling in the ischemic myocardium, with increases in both antioxidant and pro-oxidant enzymes. The changes in myocardial oxidant signaling delineated in this study likely play a critical role in pathways culminating in the effects on myocardial remodeling and function shown in our previous work [31].

## Figures and Tables

**Table 1:** Antibodies. Table 1 lists all antibodies used in the experiment, including manufacturer, catalog number, and dilution information. KEAP 1, kelch-like ECH associated protein 1; NF-κB, nuclear factor kappa-light-chain-enhancer of activated B.

Antibody	Company	Catalog Number	Dilution
Primary Antibodies			
3-Nitrotyrosine	Novus	NB110-96877	1:100
Catalase	Cell Signaling	12980	1:1000
Glutathione Peroxidase-1	Cell Signaling	3206	1:1000
Heat Shock Factor 1	Cell Signaling	12972	1:1000
Heme Oxygenase-1	Cell Signaling	43966	1:1000
KEAP-1	Proteibtech	60027	1:1000
NF-kB	Cell Signaling	8242	1:1000
NADPH Oxidase 1	Abcam	ab121009	1:2000
NADPH Oxidase 2	Abcam	ab80897	1:1000
NADPH Oxidase 3	Proteibtech	20065	1:1000
p47phox	Abcam	ab795	1:2000
p67phox	Proteibtech	67594	1:1000
Peroxiredoxin-1	Cell Signaling	8499	1:1000
Superoxide Dismutase 2	Cell Signaling	13141	1:1000
Mitochondrial Uncoupling Protein 2	Proteibtech	11081	1:1000
Secondary Antibodies			
Anti-goat HRP-linked Antibody	Abcam	ab97110	1:2000
Anti-mouse HRP-linked Antibody	Cell Signaling	7076S	1:2000
Anti-rabbit HRP-linked Antibody	Cell Signaling	7074S	1:2000

**Table 2:** Hypoxia-conditioned extracellular vesicle proteomic analysis. Proteomic analysis of the HEVs identified 395 unique proteins as previously reported by our group. This included 16 proteins with known antioxidant function. The data are represented as log base 10 of the total protein abundance in molecules.

Protein Name	Log10 (Abundance)
Annexin A1	6.2
Catalase	6.5
Ferritin Heavy Chain 1	7.3
Glutathione S-Transferase Pi 1	5.3
Heat Shock Protein B1	6.4
Hemoglobin Subunit Alpha 2	8.4
Hemoglobin Subunit Epsilon 1	6.3
Peroxiredoxin-1	6.6
Peroxiredoxin-2	5.4
Peroxiredoxin-5	4.8
Peroxiredoxin-6	6.3
Phosphoglycerate Kinase 1	5.6
Protein Disulfide-Isomerase	6.2
Ras-Related Protein Rap-1b	7.6
Superoxide Dismutase 1	5.9
Thioredoxin	4.2

**Table 3:** Myocardial prefusion correlation. Table 2 demonstrates the correlation of previously reported myocardial perfusion data with protein expression [29]. There was no significant correlation between protein expression and myocardial perfusion elicited using Spearman’s rank correlation coefficient technique (r). GPX-1, glutathione peroxidase-1; HSF-1, heat shock factor 1; HO-1, heme oxygenase-1; NOX 1, NADPH oxidase 1; NOX 3, NADPH oxidase 3; SOD 2, superoxide dismutase 2; UCP 2, mitochondrial uncoupling protein 2.

	Rest		Paced	
	r	*p*	r	*p*
Catalase	0.00	1.00	−0.04	0.96
GPX-1	−0.61	0.17	−0.14	0.78
HSF-1	−0.36	0.44	−0.21	0.66
HO-1	−0.75	0.07	0.04	0.96
NOX 1	−0.50	0.27	0.46	0.30
NOX 3	0.32	0.50	0.00	1.00
p47phox	0.00	1.00	0.21	0.66
p67phox	−0.71	0.09	0.14	0.78
SOD 2	0.64	0.14	−0.07	0.91
UCP 2	0.54	0.24	0.14	0.78

## Data Availability

Data are available upon request to the corresponding author.

## Abbreviations

Control (CON); Coronary artery disease (CAD); End-systolic pressure–volume relation (ESPVR); Extracellular vesicle (EV); Glutathione peroxidase-1 (GPX-1); Heat shock factor 1 (HSF-1); Heme oxygenase-1 (HO-1); Hypoxia-conditioned extracellular vesicles (HEVs); Kelch-like ECH associated protein 1 (KEAP 1); Left circumflex coronary artery (LCx); Marrow–derived stem cells (HBMSCs); Mitochondrial uncoupling protein 2 (UCP-2); NADPH oxidase 1 (NOX 1); NADPH oxidase 2 (NOX 2); NADPH oxidase 3 (NOX 3); Nitrotyrosine (3NT); Peroxiredoxin-1 (PRDX1); Preload recruitable stroke work (PRSW); Superoxide dismutase 2 (SOD 2)
